# Supplementary material for: Dynamics of single human embryonic stem cells and their pairs: a quantitative analysis
Source: Sci Rep. 2017 Apr 3;7:570. doi: 10.1038/s41598-017-00648-0 (PMC5428844; doi:10.1038/s41598-017-00648-0)
Supplement: Supplementary file 1 — Supplementary Information [file 41598_2017_648_MOESM1_ESM.pdf]

SUPPLEMENTARY INFORMATION

Dynamics of single human embryonic stem cells and their pairs: a quantitative analysis

L. E. Wadkin, L. F. Elliot, I. Neganova, N. G. Parker, V. Chichagova, G. Swan, A. Laude, M. Lako, and A. Shukurov

Supplementary Table T1

Table 1. Summary of parameters acquired for single cells cultured in the absence and presence of Cell Tracer. The entries represent the mean values with their standard errors and the spread within the sample given by the standard deviation (SD) of individual measurements around the mean. Migration velocities v and step lengths l_x and l_y were calculated by averaging the displacements between images taken at 15 min intervals.

Parameter and notation		No Cell Tracer		With Cell Tracer	
		Average	SD	Average	SD
Number of cells	N	26	–	22	–
Migration velocity (μm/hr)	v	22.7 ± 0.6	16.5	14.1 ± 0.4	12.2
Step length in x (μm)	l_x	3.5 ± 0.1	3.4	2.2 ± 0.1	2.4
Step length in y (μm)	l_y	3.5 ± 0.1	3.5	2.1 ± 0.1	2.5
Time to first division (hr)	t_{div}	7 ± 1	5	10 ± 2	6.5

Supplementary Table T2

Table T2. Parameters characterising the migration of hESC pairs, both in the absence and in the presence of Cell Tracer. For each parameter, we present its mean value and standard deviation, as well as the spread given by the standard deviation of the individual measurements from the mean.

Parameter and notation		No Cell Tracer				With Cell Tracer	
		Type A		Type B			
		Average	SD	Average	SD	Average	SD
Number of pairs	N	10		12		18	
Tracking time (hr)		23		24.25		31.5	
Initial separation (μm)	s_0	17 ± 1	4	18 ± 1	5	18 ± 1	6
Final separation (μm)	s_{f}	123 ± 27	87	42 ± 9.	32	19 ± 2	7
Maximum separation (μm)	$s_{\max}$	141 ± 30	95	117 ± 17	58	49 ± 2	8
Time to $s_{\max}$ (hr)	$T_{\max}$	16 ± 2	6	7 ± 1	4	7 ± 2	8
Speed of each cell (μm/hr)	v	25.8 ± 0.5	20.4	24.8 ± 0.4	17.5	17.5 ± 0.4	14.0
Pair centroid speed (μm/hr)	v_{pc}	18.7 ± 0.5	13.6	18.6 ± 0.4	12.4	14.1 ± 0.4	11.1
Relative speed (μm/hr)	v_{r}	38 ± 1	28	33.3 ± 0.8	23.7	20.8 ± 0.6	17.1

Supplementary Section S1: Directionality

Directionality, (or the straightness index) is a simple and convenient parameter to quantify an isotropic random walk, as employed by Li *et al*[1]. The displacement of the cell at a time t, measured along the straight line from the starting point is $L_i = \sqrt{[x_i(t) - x_{i,0}]^2 + [y_i(t) - y_{i,0}]^2}$ (with i the cell identifier) and the total distance traversed during the time t is denoted T_i. The directionality of the cell migration is then defined as $\Delta_i = L_i/T_i$, and its values lie in the range $0 \le \Delta_i \le 1$. If the cell moves along a straight path, we have $T_i = L_i$, and the directionality has its maximum value, $\Delta_i = 1$. If, however, the cell follows a long and tortuous trajectory, then T_i is much larger than L_i, and the directionality is low, $\Delta_i \approx 0$. Thus, the directionality quantifies how tangled and convoluted the cell's trajectory is. This quantity is closely related to the *tortuosity*, similarly characterising the shape of convoluted trajectories[2]. While

the directionality may not the most useful characteristic of trajectories[3], we use it to retain comparability with earlier work on cell kinematics[3]. In particular, the directionality depends on the number of steps taken in the random walk. However, the unstained and stained cells move with similar correlation times, performing similar number of steps per unit time; this allows us to compare their trajectories in real time. It would not be difficult to describe the trajectories in terms of the number of random walk steps, but such a description would be less intuitive.

For a two-dimensional isotropic random walk, with steps of a length l, the average displacement from the starting point increases with the number of steps N as $L_i = l\sqrt{N}$, where $N = t/\tau$ is the number of steps in time t. Meanwhile, the total distance traversed is $T_i = lN$. Then the average directionality of an isotropic random walk varies with time as[11]

$$\Delta_i \simeq \frac{1}{\sqrt{N}} = \sqrt{\frac{\tau}{t}}, \qquad (1)$$

decreasing towards zero as the number of steps N, or time t, increases. The reduction of the average directionality with time in inverse proportion to the square root of time elapsed since the start of the migration is a diagnostic property of an isotropic random walk. Note that Equation (1) gives the *averaged* directionality; the displacement and directionality for a single walker may deviate significantly due to the probabilistic nature of the walk.

To further confirm that the cell migration for unstained cells is consistent, on average, with the theory of isotropic random walk, we consider the average directionality (averaged over all 26 single hESCs) versus time, shown in Figure S1a. Up to around 7 hours there is a systematic decrease in the averaged directionality from unity to low values, in qualitative agreement with the random walk behaviour. To ascertain the functional form of this decay, the data is plotted on log-log axes (inset of Figure S1a). The prominent straight-line behaviour during this time indicates that the directionality decays as a power-law with time, and a straight-line least-squares fit (not constrained to go through any particular point) gives $\overline{\Delta_\iota}(t) = (0.50 \pm 0.02)t^{-0.44\pm0.04}$. The scaling with time is close to the $t^{-1/2}$ dependence characteristic of the isotropic random walk, Equation (1). Beyond 7 hours, the evolution of the average directionality changes its character and deviates from the $1/\sqrt{t}$ random walk behaviour; a similar deviation was noted in the plot of mean-square displacement versus time in Figure 3.

The averaged directionality in the presence of Cell Tracer, shown in Figure S1b, also indicates the systematic decrease over time, characteristic of an isotropic random walk. The least-squares fit is $\overline{\Delta_\iota}(t) = (0.61 \pm 0.05)t^{-0.50\pm0.04}$, which is also close to the $t^{-1/2}$ scaling characteristic of the diffusive motion.

The directionality of the unstained pair centroid motion, shown in Figure S2a, confirms that the pair as a whole can be described as an unbiased random walk at a good level of accuracy over the observation time range. The least-squares fit for unstained cells $\overline{\Delta_\iota}(t) = (0.49 \pm 0.02)t^{-0.42\pm0.02}$. The pair centroid motion of Cell Tracer stained cells is also consistent with a random walk: the least-squares fit is $\overline{\Delta_\iota}(t) = (0.54 \pm 0.06)t^{-0.56\pm0.06}$, shown in Figure S2b.

Supplementary Figure S1

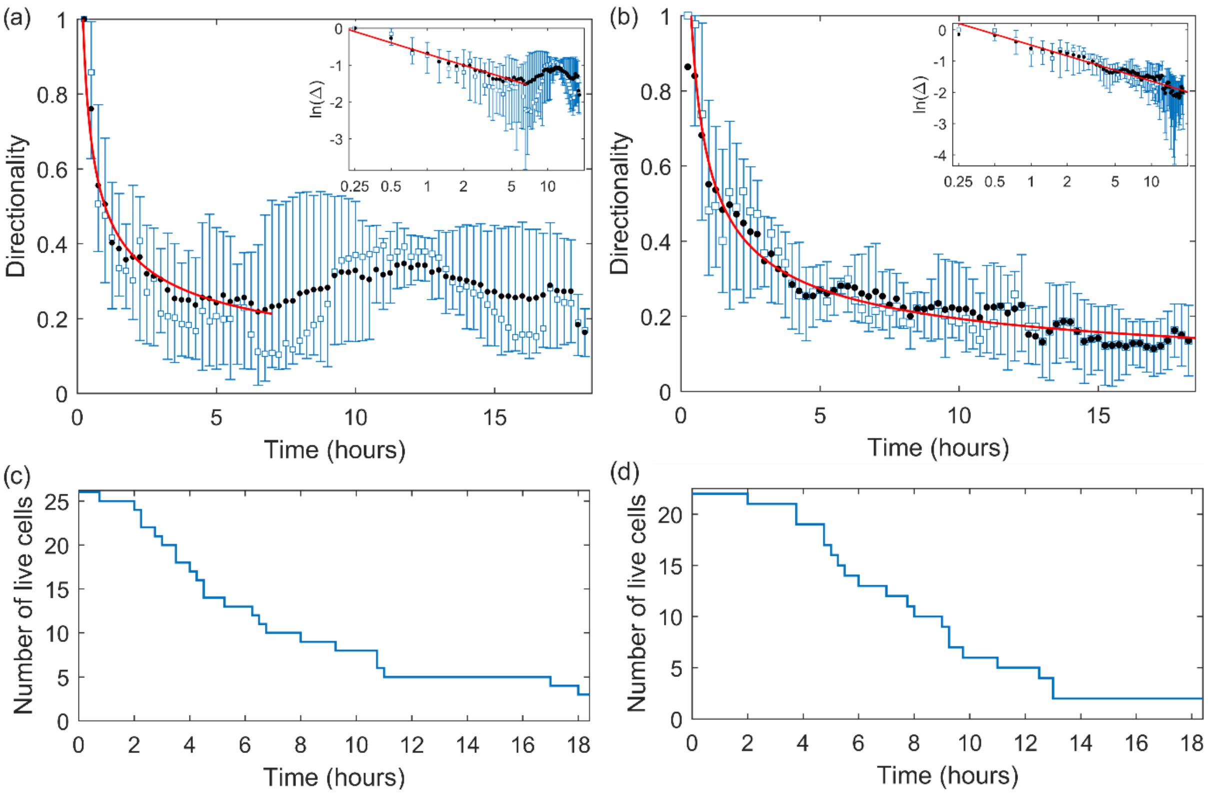

Supplementary Figure S1: Mean (black circles) and median (blue squares) directionality over time for the migration of single hESCs in (a) the absence of Cell Tracer, and (b) the presence of Cell Tracer. Insets show the data on natural logarithmic axes. Straight lines are a least-squares fit, applied to the whole time range in (b) and up to 7 hours in (a). These fits are (a) $\overline{\Delta_\iota}(t) = (0.50 \pm 0.02)t^{-0.44\pm0.04}$ and (b) $\overline{\Delta_\iota}(t) = (0.61 \pm 0.05)t^{-0.50\pm0.04}$. Error bars show the upper and lower quartiles. The number of live cells over time for unstained cells (c) and stained cells (d) to indicate the changing sample size. The sampling interval is every 15 minutes.

Supplementary Figure S2

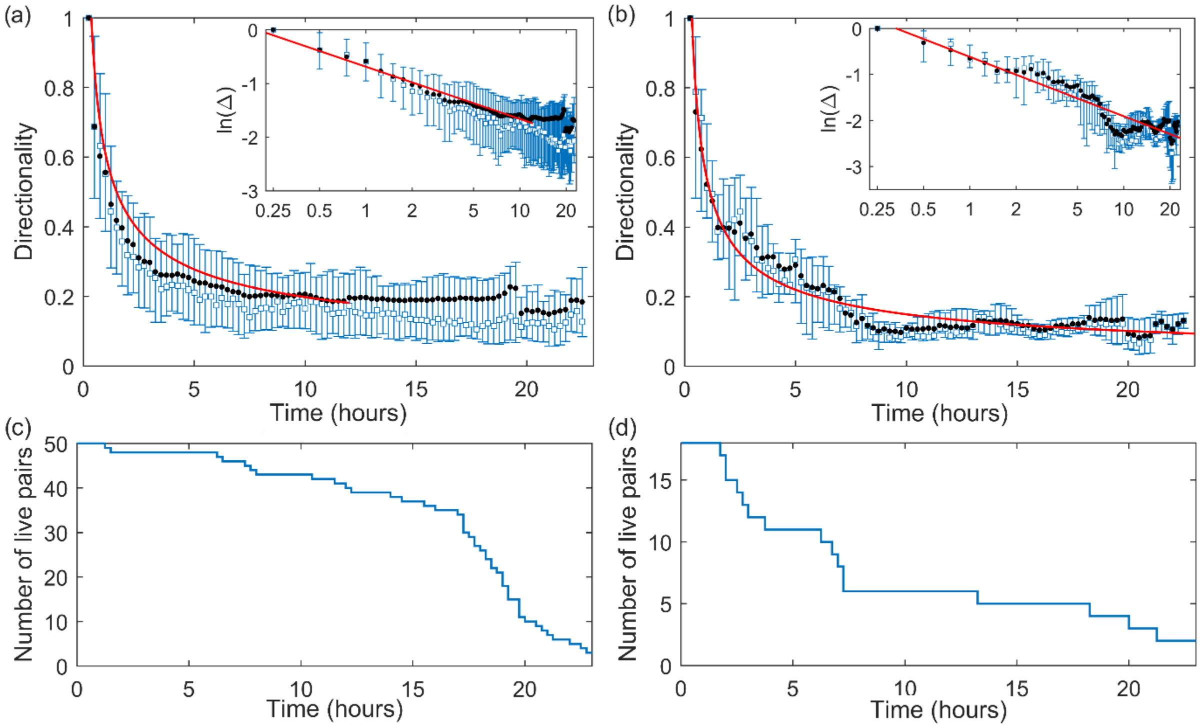

Supplementary Figure S2. Mean (black circles) and median (blue squares) directionality over time for the migration of pairs of hESCs in (a) the absence of Cell Tracer, and (b) the presence of Cell Tracer. Insets show the data on natural logarithmic axes. Straight lines are a least-squares fit, applied to the whole time range in (b) and up to 12 hours in (a). These fits are (a) $\overline{\Delta}_\iota(t) = (0.49 \pm 0.02)t^{-0.42\pm0.02}$ and (b) $\overline{\Delta}_\iota(t) = (0.54 \pm 0.06)t^{-0.56\pm0.06}$. Error bars show the upper and lower quartiles. The number of live cells over time for unstained cells (c) and stained cells (d) to indicate the changing sample size. The sampling interval is every 15 minutes.

Supplementary Figure S3

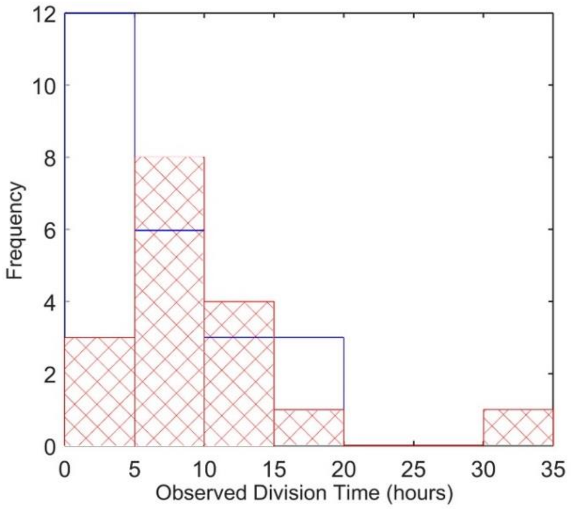

Supplementary Figure S3: Histograms of division times, with bin widths of 5 hours in each case, for the single hESCs in the absence (blue) and presence of Cell Tracer (cross hatched in red). The Kolmogorov–Smirnov two-sample test confirms that the two distributions are distinct, suggesting that the Cell Tracer treatment affects significantly the ability of the cells to divide. The Mann-Whitney U test also confirms the two distributions are distinct ($p < 0.05$).

Supplementary Figure S4

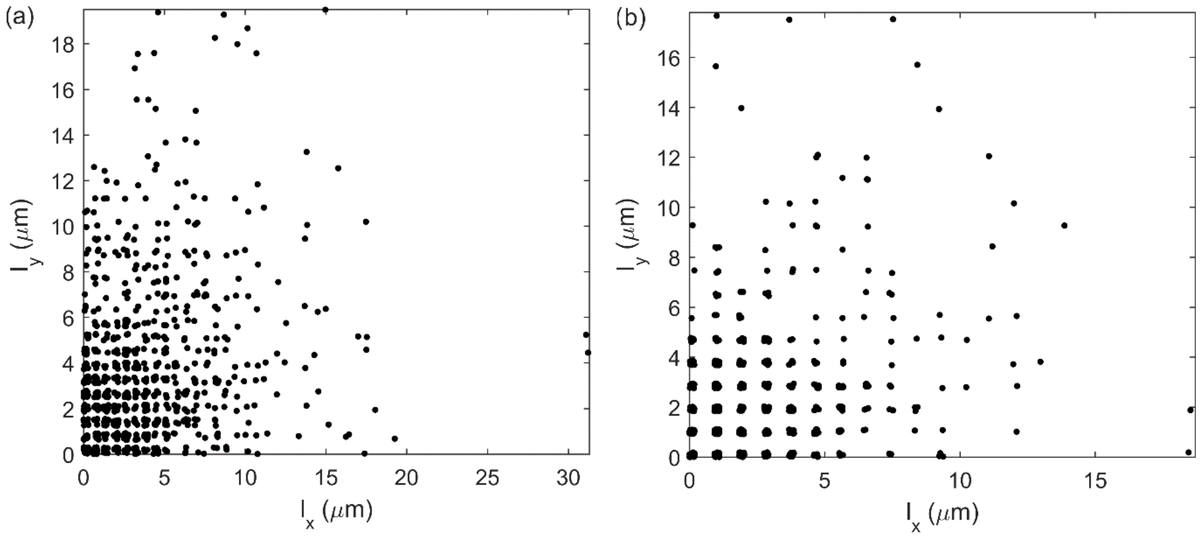

Supplementary Figure S4: The scatter plot of the step lengths l_x and l_y at each time frame (every 15 minutes) for cells (a) without Cell Tracer and (b) with Cell Tracer. Together with the low cross-correlation coefficient between the two variables discussed in the main text, the lack of any pronounced correlation between l_x and l_y [except perhaps the rare events with large values of l_y in Panel (a)] suggests the isotropy of the random walk. According to the Kolmogorov-Smirnov and Mann-Whitney U tests, there is no evidence to distinguish between the distributions of l_x and l_y. The Pearson product-moment correlation coefficient of l_x and l_y is as small as 0.22, confirming the steps in the x and y directions are uncorrelated.

Supplementary Figure S5

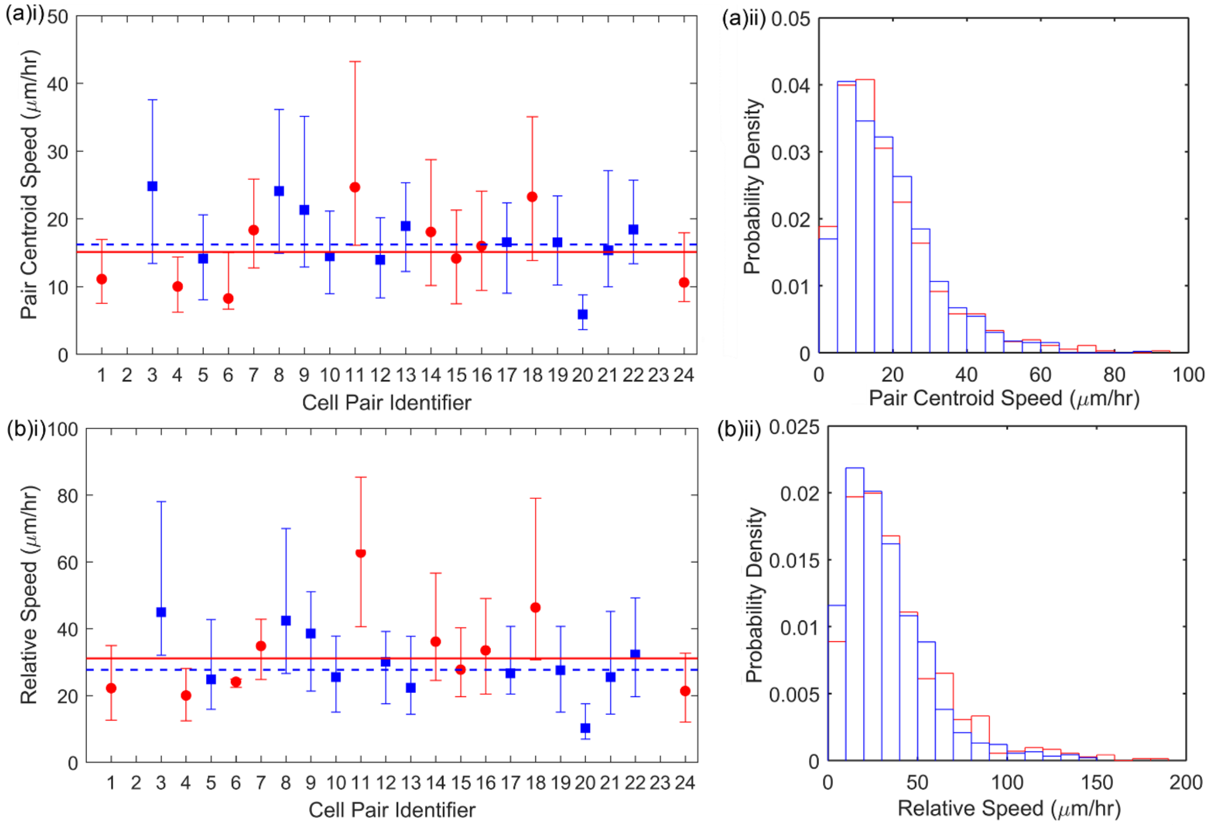

Supplementary Figure S5: (a) The speed of the pair centroid in the absence of Cell Tracer for the Type A (red) and Type B (blue) pairs: (i) the median speeds, with error bars representing the upper and lower quartiles, and (ii) the corresponding probability densities of the centroid speeds. Horizontal lines in (i) indicate the average across the entire category. (b): as in Panels (a) but for the relative speed within a pair. According to Kolmogorov–Smirnov and Mann-Whitney U tests the probability distributions for the Type A and Type B relative speeds are different.

Supplementary Figure S6

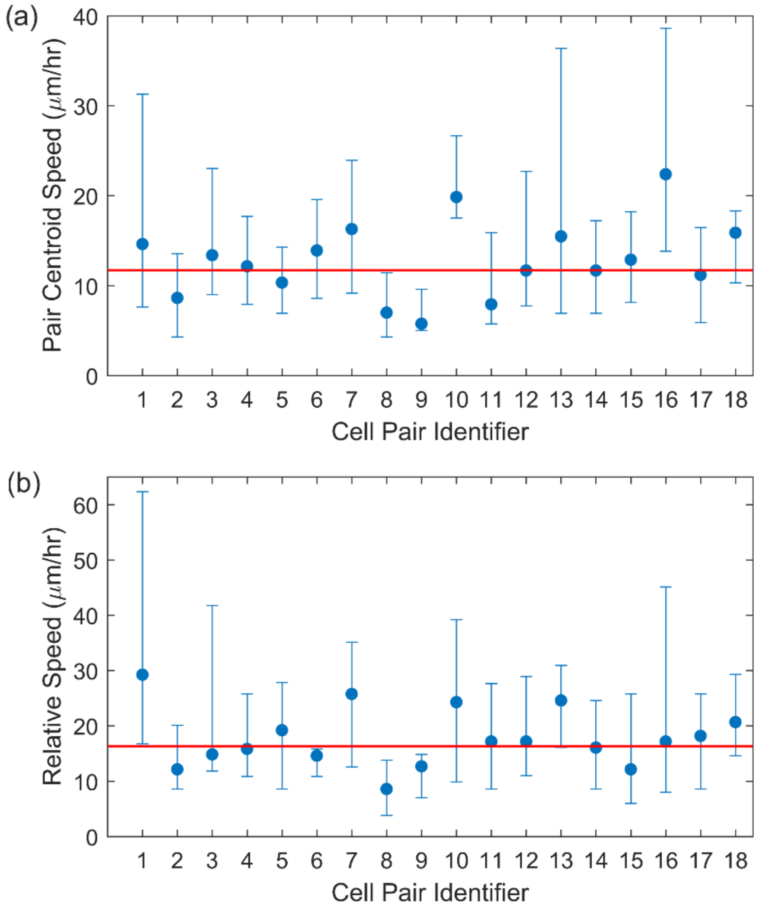

Supplementary Figure S6. (a) Median pair centroid speeds and (b) relative speeds of cell pairs in the presence of Cell Tracer.

Supplementary Figure S7

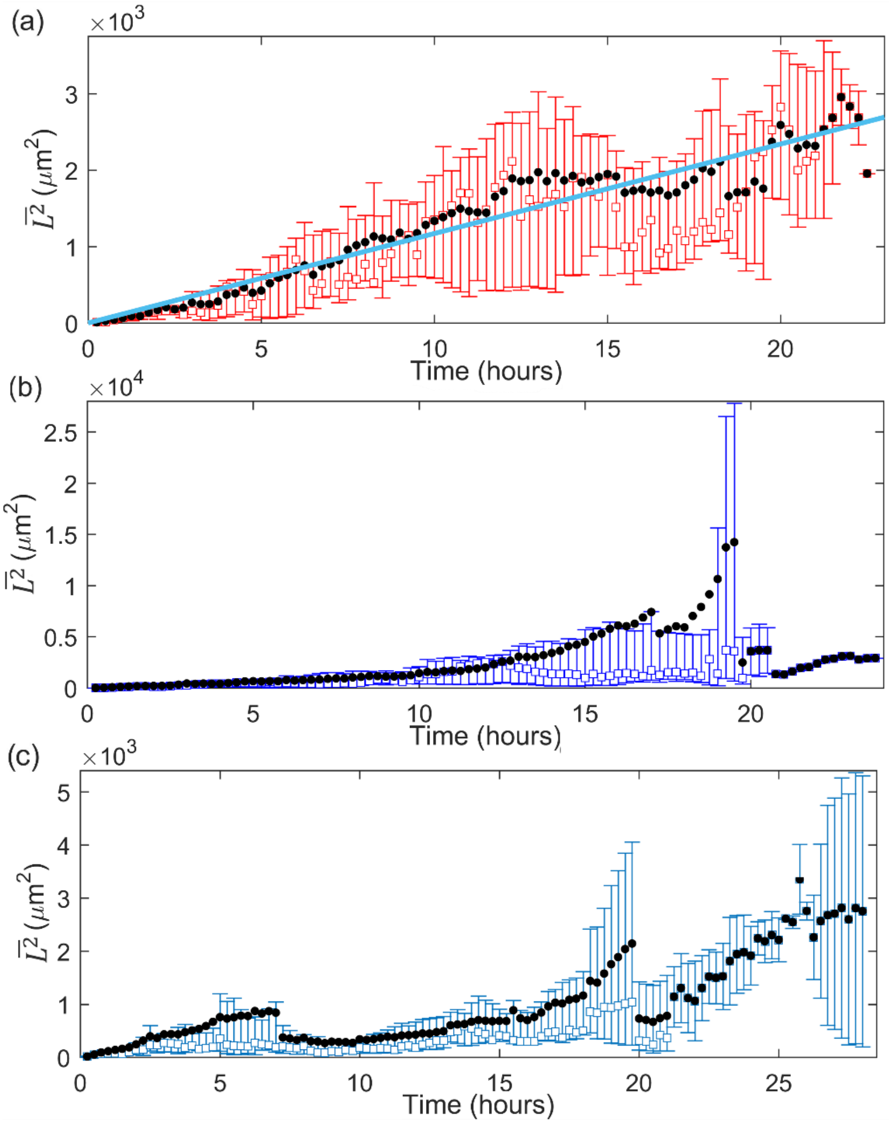

Supplementary Figure S7: The time evolution of the mean (black circles) and median (squares) centroid L^2 for pairs of unstained cells of Type A (a) and unstained cells of Type B (b) and stained cells (c). The least-squares fit for Type A cells is $L^2 = 2Dt$ with $D = 58.45 \pm 1.8$ μm^2/hr. Error bars show the upper and lower quartiles. The sampling interval is 15 minutes.

Supplementary Section S3: Correlated Random Walk

An alternative model for the migration of the cells is a correlated random walk, where the direction of a new step depends on the direction of the previous step, so that the migration retains a short-term memory of its direction[10]. We recall that the direction of movement is selected independently of the previous direction in the ordinary random walk. The migration remains diffusive over long time and length scales, but the diffusivity now depends on the mean value of the cosine of the angle θ between the two consecutive displacements, denoted here $\langle\cos\theta\rangle$:

$$D = \frac{\langle l^2\rangle}{2\tau} + \frac{\langle l\rangle^2\langle\cos\theta\rangle}{\tau(1-\langle\cos\theta\rangle)},$$

where, as before, τ is the correlation time, $\langle l^2\rangle$ and $\langle l\rangle$ are the mean squared length of the steps and their mean length, respectively, and angular brackets denote averaging[12]. For $\langle\cos\theta\rangle = 0$ (e.g., for θ uniformly distributed between 0 and 2π), the standard expression (3) is recovered, with $\langle l^2\rangle = \langle v^2\rangle/\tau$. For $\langle\cos\theta\rangle \to 1$ (unidirectional motion), $D \to \infty$ signifying a non-diffusive motion.

For a correlated random walk, the relation of the correlation time to the diffusivity changes from $\tau = 2D/\langle v^2\rangle = \langle l^2\rangle/(2D)$, used in the main text, to

$$\tau = \frac{\langle l^2\rangle/2 + \langle l\rangle^2\langle\cos\theta\rangle/(1-\langle\cos\theta\rangle)}{D}.$$

To assess possible importance of the short-time correlations, consider two cases that illustrate the range of possibilities, assuming $\langle l^2\rangle = \langle l\rangle^2$ (e.g., a constant step length l). The diffusion coefficient obtained for an uncorrelated random walk is $D_0 = l^2/(2\tau)$. For $\langle\cos\theta\rangle = 1/2$, we obtain $D = 3D_0$, and the correlation time derived as $\tau = l^2/(2D)$ would is three times longer than its true value $\tau = l^2(1 + \langle\cos\theta\rangle)/[2D(1-\langle\cos\theta\rangle)]$. Alternatively, for $\langle\cos\theta\rangle = -1/2$, we have $D = D_0/3$ and the correlation time inferred using the assumption of uncorrelated random walk is three times shorter than the true value.

We have not noticed any obvious signs that would suggest that the cell migration is better modelled as a correlated random walk. Since the correlation time of the cell migration is not affected much by the staining, the comparisons and conclusions discussed in the text are independent of this aspect of the random walk, if the staining only affects the parameters of the random walk (as we assume) rather than destroys or introduces any significant short-time correlations. However, this interesting question deserves further careful analysis.

---

[1] Li, L., et al. Individual Cell Movement, Asymmetric Colony Expansion, Rho-Associated Kinase, and E-Cadherin Impact the Clonogenicity of Human Embryonic Stem Cells, *Biophys. J.* **98**: 2442–2451 (2010).

[2] Benhamou, S. How to reliably estimate the tortuosity of an animal's path: straightness, sinuosity, or fractal dimension? *J. Theor. Biol.* **229**, 209–220 (2004).

[3] Codling, E. A., Plank, M. J. and Benhamou, S. Random walk models in biology. *J. R. Soc. Interface*: **5**, 813 (2008).
